# Adipose tissue gene expression analysis reveals changes in inflammatory, mitochondrial respiratory and lipid metabolic pathways in obese insulin-resistant subjects

**DOI:** 10.1186/1755-8794-5-9

**Published:** 2012-04-03

**Authors:** Jarkko Soronen, Pirkka-Pekka Laurila, Jussi Naukkarinen, Ida Surakka, Samuli Ripatti, Matti Jauhiainen, Vesa M Olkkonen, Hannele Yki-Järvinen

**Affiliations:** 1 FIMM, Institute for Molecular Medicine Finland, University of Helsinki, Tukholmankatu 8, Helsinki 00290, Finland; 2 Public Health Genomics Unit, National Institute for Health and Welfare, Haartmaninkatu 8, Helsinki 00290, Finland; 3 Department of Medicine, Division of Diabetes, Helsinki University Central Hospital, Haartmaninkatu 8, Helsinki 00290, Finland; 4 Minerva Foundation Institute for Medical Research, Tukholmankatu 8, Helsinki 00290, Finland

## Abstract

**Background:**

To get insight into molecular mechanisms underlying insulin resistance, we compared acute in vivo effects of insulin on adipose tissue transcriptional profiles between obese insulin-resistant and lean insulin-sensitive women.

**Methods:**

Subcutaneous adipose tissue biopsies were obtained before and after 3 and 6 hours of intravenously maintained euglycemic hyperinsulinemia from 9 insulin-resistant and 11 insulin-sensitive females. Gene expression was measured using Affymetrix HG U133 Plus 2 microarrays and qRT-PCR. Microarray data and pathway analyses were performed with Chipster v1.4.2 and by using in-house developed nonparametric pathway analysis software.

**Results:**

The most prominent difference in gene expression of the insulin-resistant group during hyperinsulinemia was reduced transcription of nuclear genes involved in mitochondrial respiration (mitochondrial respiratory chain, GO:0001934). Inflammatory pathways with complement components (inflammatory response, GO:0006954) and cytokines (chemotaxis, GO:0042330) were strongly up-regulated in insulin-resistant as compared to insulin-sensitive subjects both before and during hyperinsulinemia. Furthermore, differences were observed in genes contributing to fatty acid, cholesterol and triglyceride metabolism (FATP2, ELOVL6, PNPLA3, SREBF1) and in genes involved in regulating lipolysis (ANGPTL4) between the insulin-resistant and -sensitive subjects especially during hyperinsulinemia.

**Conclusions:**

The major finding of this study was lower expression of mitochondrial respiratory pathway and defective induction of lipid metabolism pathways by insulin in insulin-resistant subjects. Moreover, the study reveals several novel genes whose aberrant regulation is associated with the obese insulin-resistant phenotype.

## Background

Instead of merely being a storage depot, adipose tissue is now recognized as an important regulator of energy homeostasis and a central player in the development of insulin resistance [1,2]. The development of insulin resistance is closely associated with obesity [2]. However, it is not known why all obese subjects are not insulin-resistant and insulin resistance can also be observed in lean subjects, highlighting the fact that the molecular mechanisms underlying insulin resistance are still largely unknown.

A number of transcriptome studies have reported differences in adipose tissue mRNA expression at fasting state between obese and lean subjects (reviewed in [3]). Most of these investigations have been performed after an overnight fast [4-14] and they compare differences between obese and non-obese subjects [5,6,10-14], predominantly in males or mixed groups consisting of both males and females.

A number of studies employing different set-ups, including global transcriptome analyses, have implicated low-grade adipose tissue inflammation as one of the central factors involved in the development of insulin resistance [15]. As an example, analysis of subcutaneous adipose tissue transcript profiles in monozygotic twins revealed elevated expression of several pro-inflammatory cytokines and complement components in the in the obese, insulin-resistant co-twins as compared to the non-obese ones [12].

Using human whole genome microarrays, Dahlman et al. [16] observed down-regulation of mitochondrial electron transport chain genes in both visceral and subcutaneous adipose tissue of type 2 diabetic subjects during fasting. A study on monozygotic twins discordant for BMI reported decreased branched-chain amino acid catabolism and reduced mitochondrial DNA copy number in the adipose tissue of obese co-twins, further emphasizing a role for mitochondrial energy and amino acid metabolism in obesity and insulin resistance [12].

Baranova et al. [4] reported mRNA expression patterns in visceral adipose tissue of morbidly obese patients compared with non-obese controls. The differentially expressed genes included those related to lipid and glucose metabolism, membrane transport, and promotion of the cell cycle. Using isolated adipocytes, Yang et al. [14] discovered through comparison of gene expression between non-diabetic insulin-resistant first degree relatives of T2D patients and healthy controls, that insulin resistance is associated with impaired adipogenesis.

While previous studies have identified a number of factors involved in adipogenesis, adipose tissue lipid and energy metabolism, as well as in inflammatory processes to be abnormally expressed, the alterations could be unrelated to insulin action and merely reflect the impact of factors other than insulin on gene expression in obese as compared to non-obese subjects. To relate the abnormalities to impaired insulin response, comparison of differences in acute regulation of the transcriptome by insulin between obese and non-obese subjects would seem of interest. To our knowledge there are no such data available. Therefore the present study was undertaken to compare transcriptional differences in subcutaneous adipose tissue derived from obese insulin-resistant and lean insulin-sensitive women before and during euglycemic hyperinsulinemia.

## Methods

### Ethics statement

The nature and potential risks of the study were explained to all subjects prior to obtaining their written informed consent. The study was carried out in accordance with the principles of the declaration of Helsinki. The protocol was approved by the ethics committee of the Helsinki University Central Hospital.

### Study subjects

A total of 20 non-diabetic Caucasian women were recruited based on the following inclusion criteria: 1) age 18-60 years; 2) no known acute or chronic disease other than obesity based on history, physical examination and standard laboratory tests (blood counts, serum creatinine, thyroid stimulating hormone, electrolyte concentrations, and electrocardiogram); and 3) body mass index (BMI) < 40 kg/m^2^. Other exclusion criteria included pregnancy or treatment with drugs that may alter glucose tolerance. Data on expression of some individual genes from these women have previously been published [17,18]. Whole body insulin sensitivity was measured in each subject using the euglycemic hyperinsulinemic clamp technique, as described [19]. The duration of the insulin infusion was 6 hours and insulin infusion rate 1 mU/kg·min.

Metabolic studies were performed after an overnight fast. Two 18-gauge catheters (Venflon; Viggo-Spectramed, Helsingborg, Sweden) were inserted, one in an antecubital vein for infusion of insulin (Insulin Actrapid; Novo Nordisk, Denmark) and glucose, and another retrogradely in a heated hand vein to obtain arterialized venous blood for measurement of glucose concentrations every 5 min and serum free insulin concentration every 30 min. The rate of the continuous insulin infusion was 1 mU kg - 1 min - 1 for 6 h. Normoglycemia was maintained by adjusting the rate of a 20% glucose infusion based on plasma glucose measurements from arterialized venous blood every 5 min. Wholebody insulin sensitivity was determined from the glucose infusion rate required to maintain normoglycemia between 30 and 360 min [19].

### Other measurements

Blood samples were taken after an overnight fast for measurement of plasma glucose, serum insulin, C-peptide, serum triglyceride and total and HDL cholesterol concentrations, as described [17].

### Adipose tissue biopsies, total RNA preparation and microarray processing

Needle aspiration biopsies of abdominal subcutaneous fat were taken under local intracutaneous anesthesia at baseline (0 h) and after 3 and 6 hours of hyperinsulinemia from the left and right lower abdominal region [20] and frozen in liquid nitrogen until analysis. Total RNA was prepared from frozen fat tissue (on the average 250 mg) using the RNeasy Lipid Tissue Mini Kit (Qiagen) according to the manufacturer's protocol. Quality of RNA was analyzed using the 2100 Bioanalyzer platform (Agilent Technologies). For microarray gene expression analysis, the five most insulin-sensitive and the five most insulin-resistant patients were selected based on their whole body insulin sensitivity (M-value). Two micrograms of total RNA from time point 0 h (fasting) and 3 h (hyperinsulinemia) were processed according to the conventional Affymetrix eukaryotic RNA labeling protocols (Affymetrix, Santa Clara, CA). Hybridization, staining and washing of the Affymetrix U133 Plus 2.0 chips were performed using the Affymetrix Fluidics Station 450 and Hybridization Oven 640 under standard conditions.

### Pathway analysis

Analysis of the microarray expression data was done using the R/Bioconductor through a graphical user interface, Chipster (v1.4.3, CSC, Finland, http://chipster.csc.fi/). The microarray data has been submitted to GEO database (accession number, GSE26637). Pre-processing of the expression data was performed using GC-RMA algorithm and re-annotated probe set libraries by the University of Michigan http://brainarray.mbni.med.umich.edu. Expression data were filtered before pathway analysis by flags. All probes with status 'present' or 'marginal' in at least 3 out of 5 chips in one of the four condition groups (fasted insulin-sensitive, fasted insulin-resistant, hyperinsulinemic insulin-sensitive, hyperinsulinemic insulin-resistant) were accepted for analysis. Probes passing these criteria were then divided into up and down-regulated probes by their fold change in expression. Analysis was done separately for fasting samples (fasted insulin-resistant/fasted insulin sensitive fold change) and for hyperinsulinemia samples (hyperinsulinemic insulin-resistant/hyperinsulinemic insulin-sensitive fold change). Probe lists were ranked according to statistical significance (Student's *t*-test) and submitted to the pathway analysis software. The top 10 up and down-regulated pathways are reported. Similarly, we analyzed which biological pathways show the strongest response in gene expression after systemic stimulation by insulin. Analyses were performed separately for the insulin-resistant and the insulin-sensitive groups (hyperinsulinemic insulin-sensitive/fasted insulin-sensitive fold change and hyperinsulinemic insulin-resistant/fasted insulin-resistant fold change). After defining the most insulin responsive pathways, we selected two of them, and compared their expression between the study groups during hyperinsulinemia. Pathway analysis was performed using an in-house developed non-parametric pathway analysis software to resolve which gene sets from GO classification are enriched in a list of genes as described recently [12]. As a feature of the dendrogram-like structure of the GO classifications, gene sets get progressively larger and less descriptive when moving down the tree. Therefore, in order to concentrate on the biologically more meaningful pathway collections, an arbitrary cut-off of a maximum of 150 genes per pathway was chosen as the largest reported gene list. In addition, all pathways referring to cellular localization were removed.

### List of differentially expressed genes

Expression data was filtered with stringent criteria to reveal only the most significant differentially expressed probes from the dataset. Every probe with flag status 'present' was accepted and the data were further filtered by standard deviation (95% filtered out). Statistical analysis was performed with 2-way analysis of variance (ANOVA), using R-statistics, and taking into account the repeated measures from the same subjects (Additional file 1). All probes with p < 0.05 after Benjamini-Hochberg multiple testing correction [21] were accepted. Two supplement tables were generated: one for significantly regulated probes between the groups (insulin-resistant vs. insulin-sensitive) and a second one for significantly regulated probes by insulin treatment (fasting vs. hyperinsulinemia).

### Quantitative RT-PCR

Quantitative RT-PCR (qPCR) was used to measure the relative abundance of interesting transcripts found from the microarray analysis. Samples from 20 women, including the 10 samples used for the microarray analysis, at three time points (0 h, 3 h and 6 h) were analyzed. Reverse transcription was performed using M-MLV reverse transcriptase (Invitrogen) using oligo(dT)12-18 primers. Power SYBR^® ^Green PCR Master Mix and ABI 7900 HT system (Applied biosystems) were used for quantitative PCR analysis with gene specific primers (Additional file 2: Table S1^Q1^) and 1 ng of template in a final reaction volume of 10 μl. Genorm algorithm [22] was used to validate three different control genes for normalization. Data were normalized to geometric mean of two control genes, beta actin (ACTB) and large ribosomal protein 0 (RPLP0).

### Statistical analyses

Clinical and biochemical characteristics in the insulin-sensitive and the insulin-resistant groups were compared using the Mann-Whitney *U *test and 2-way ANOVA. The calculations were performed with SPSS statistics 18.0 for Windows (SPSS, Chicago, IL). All data are shown as mean ± standard error of mean.

## Results

### Clinical characteristics of the study subjects

The clinical characteristics of the study subjects are summarized in Table 1. The characteristics are reported separately for subjects in the microarray study and for the subjects in the qRT-PCR experiment. Markers of insulin resistance, serum fasting insulin and C-peptide concentrations were higher, and HDL-cholesterol concentration lower in the insulin-resistant group. During insulin infusion, serum insulin concentrations were similar between the lean insulin-sensitive and the obese insulin-resistant groups (68 ± 6 vs. 84 ± 10 mU/l, respectively; NS). By definition, whole-body insulin sensitivity was 131% higher in the insulin-sensitive than the insulin-resistant group in the microarray experiment (n = 10) (9.7 ± 0.5 vs. 4.2 ± 0.4 mg kg - 1 min - 1, p < 0.009) and 107% higher in the entire study group (n = 20) (8.7 ± 0.4 vs. 4.2 ± 0.4 mg kg 1 min 1, p < 0.0001). Moreover, all indicators of obesity (body weight, BMI, whole-body-fat, fat mass, and waist-to-hip ratio were significantly higher in the insulin-resistant subjects than in the sensitive ones.

**Table 1:** Physical and biochemical characteristics of the study subjects divided into insulin-sensitive and -resistant groups on the basis of their median whole-body insulin sensitivity

	Microarray			qPCR		
	
	Insulin Sensitive	Insulin Resistant	*p *value	Insulin Sensitive	Insulin Resistant	*p*value
n	5	5	-	11	9	-

Age (years)	33 ± 6	39 ± 5	NS	32 ± 4	39 ± 3	NS

Body weight (kg)	60 ± 4	90 ± 5	0.009	69 ± 4	90 ± 5	0.003

BMI (kg/m^2^)	22.0 ± 0.7	32.5 ± 1.7	0.009	24.7 ± 1.1	33.0 ± 2.0	0.002

Whole-body fat (%)	24 ± 3	36 ± 1	0.009	28 ± 2	36 ± 1	0.002

Fat mass (kg)	15 ± 2	33 ± 3	0.009	20 ± 2	35 ± 4	0.003

Waist-to-hip ratio	0.86 ± 0.02	0.93 ± 0.02	0.047	0.86 ± 0.01	0.91 ± 0.01	0.03

fP-glucose (mmol/l)	5.0 ± 0.1	5.7 ± 0.2	0.016	5.1 ± 0.1	5.6 ± 0.2	0.012

fS-insulin (mU/l)	3 ± 0.5	11 ± 2	0.009	3 ± 0.4	10 ± 1	< 0.001

fS-LDL cholesterol (mmol/l)	2.2 ± 0.2	3.1 ± 0.2	0.016	2.2 ± 0.2	3.1 ± 0.2	0.004

fS-triglycerides (mmol/l)	0.9 ± 0.2	1.3 ± 0.2	0.076	0.78 ± 0.1	1.4 ± 0.2	0.002

fS-HDL cholesterol (mmol/l)	1.9 ± 0.09	1.4 ± 0.05	0.009	1.8 ± 0.08	1.4 ± 0.09	0.003

fS-C-peptide (nmol/l)	0.4 ± 0.03	0.8 ± 0.01	0.009	0.4 ± 0.03	0.8 ± 0.08	0.002

Data are mean ± SEM, fP = fasting plasma, fS = fasting serum

### Inflammatory pathways are up-regulated in the insulin-resistant women

**Figure 1:**
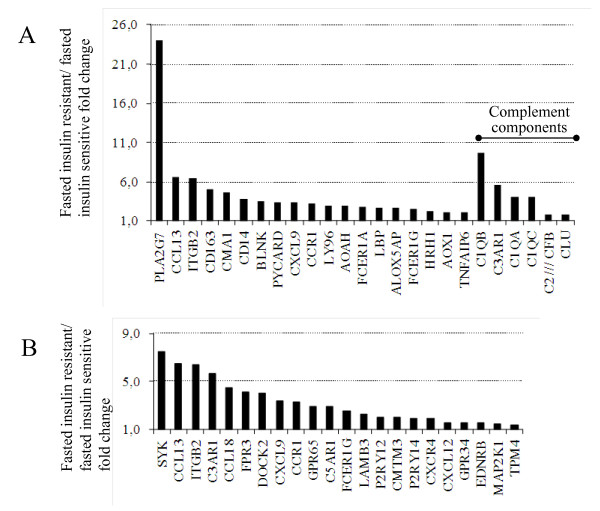
**Inflammatory response and chemotaxis pathways were up-regulated in insulin-resistant subjects**. The x-axis shows the fold change of gene expression in the inflammatory response (GO:0006954) (A) and chemotaxis pathways (GO:0006935) (B) under fasting conditions in the insulin-resistant compared to the insulin-sensitive subjects. Genes encoding complement components are indicated.

Activation of inflammatory pathways was a predominant phenotype in the insulin-resistant group after overnight fast (Table 2). The inflammatory gene expression pattern consisted mainly of two aspects of the immune response: genes involved in complement activation (inflammatory response, GO:0006954, p = 2.0E-10) and those involved in cellular chemotactic activity (taxis/chemotaxis, GO:0006935, p = 1.8E-07). The inflammatory response pathway included complement 1q subcomponent, B chain (*C1QB*), A chain (*C1QA*) and C chain (*C1QC*). In this pathway, the gene most strongly elevated in the insulin-resistant group was LDL-associated phospholipase A2 (*PLA2G7 *or *LDL-PLA2*) (Figure 1A).

**Table 2:** Ten most up-regulated pathways in insulin-resistant compared to insulin-sensitive group after an overnight fast

GO category	Pathway name	Nominal *p*	Permuted *p*
GO:0006954	inflammatory response	2.0E-10	0.0001

GO:0007626	locomotory behavior	2.8E-09	0.0001

GO:0007610	behavior	4.5E-09	0.0001

GO:0002682	regulation of immune system process	4.1E-08	0.0001

GO:0050778	positive regulation of immune response	1.0E-07	0.0001

GO:0055080	cation homeostasis	1.4E-07	0.0001

GO:0002253	activation of immune response	1.5E-07	0.0001

GO:0006968	cellular defense response	1.6E-07	0.0001

GO:0042330	taxis/chemotaxis	1.8E-07	0.0001

GO:0002684	positive regulation of immune system process	2.3E-07	0.0001

The second aspect of the inflammatory phenotype involved a number of chemokines and adhesion molecules in the taxis/chemotaxis pathways. The most prominently elevated genes in the insulin-resistant subjects were the spleen tyrosine kinase (*SYK*), chemokine *CCL13*, and beta 2 integrin (*ITGB2*) involved in leukocyte adhesion (Figure 1B). Up-regulation of inflammatory pathways similar to those in the fasted situation was observed between the groups during hyperinsulinemia: The most divergent pathways here included inflammatory response (GO:0006954, p = 3.7E-07) and regulation of immune system process (GO:0002682, p = 1.0E-06) (Table 3). Expression of marcrophage marker CD36 was 14.2% increased in the insulin-resistant group.

**Table 3:** Ten most up-regulated pathways in insulin-resistant compared to insulin-sensitive group during euglycemic hyperinsulinemic conditions

GO category	Pathway name	Nominal *p*	Permuted *p*
GO:0032787	monocarboxylic acid metabolic process	5.6E-06	0.0001

GO:0055114	oxidation reduction	9.7E-06	0.0003

GO:0006066	cellular alcohol metabolic process	1.4E-05	0.0005

GO:0008131	amine oxidase activity	2.6E-05	0.0002

GO:0006631	fatty acid metabolic process	5.8E-05	0.0012

GO:0016638	oxidoreductase activity, acting on the CH-NH2 group of donors	1.0E-04	0.0003

GO:0006725	cellular aromatic compound metabolic process	1.2E-04	0.0022

GO:0006642	triacylglycerol mobilization	1.5E-04	0.0001

GO:0042594	response to starvation	2.1E-04	0.0012

GO:0016645	oxidoreductase activity, acting on the CH-NH group of donors	2.5E-04	0.002

ANOVA analysis of single genes expressed differentially between the groups revealed additional genes of interest such as osteopontin (*SPP1*), matrix metalloproteinase 9 (*MMP9*), branched chain amino-acid transaminase 1 (*BCAT1*) and EGF-like protein 6 (*EGFL6*). These were expressed at higher levels in the insulin-resistant than the insulin-sensitive group (Additional file 3: Table S2).

### Monocarboxylic acid metabolism is down-regulated in the insulin-resistant women during fasting

**Figure 2:**
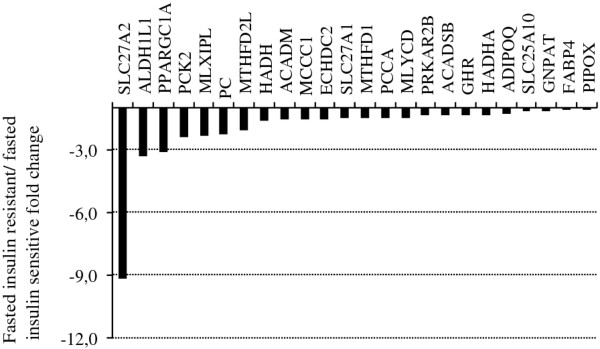
**Monocarboxylic acid metabolic process pathway was down-regulated in insulin-resistant subjects**. The x-axis shows the fold change of gene expression in the monocarboxylic acid metabolic process pathway (GO:0032787) under fasting conditions in the insulin-resistant compared to the insulin-sensitive subjects.

Expression of genes involved in monocarboxylic acid metabolism (GO:0032787, p = 5.6E-06), most of which represent the fatty acid metabolism pathway (GO:0006631, p = 5.8E-05), was reduced in the insulin-resistant group as compared with the insulin-sensitive group after an overnight fast (Table 4). Fatty acid transporter 2 (*SLC27A2 *or *FATP2*) was the most differentially expressed gene in this pathway (Figure 2). In addition, decreased mRNA expression of the oxidation reduction (GO:0055114) and cellular alcohol metabolic (GO:0006066) pathways was detected in the insulin-resistant group (Table 4).

**Table 4:** Ten most down-regulated pathways in insulin-resistant compared to insulin-sensitive group after an overnight fast

GO category	Pathway name	Nominal *p*	Permuted *p*
GO:0001934	positive regulation of protein amino acid phosphorylation	6.70E-08	0.0001

GO:0000904	cell morphogenesis during differentiation	1.62E-07	0.0001

GO:0048675	axon extension	2.63E-07	0.0001

GO:0006954	inflammatory response	3.71E-07	0.0001

GO:0005764	lysosome	6.78E-07	0.0001

GO:0010562	positive regulation of phosphorus metabolic process	7.14E-07	0.0001

GO:0005773	vacuole	7.18E-07	0.0001

GO:0002682	regulation of immune system process	1.02E-06	0.0001

GO:0006955	immune response	1.6E-07	0.0001

GO:0001932	regulation of protein amino acid phosphorylation	1.8E-07	0.0001

### Defective mRNA expression of nuclear mitochondrial genes in insulin-resistant women during hyperinsulinemia

**Figure 3:**
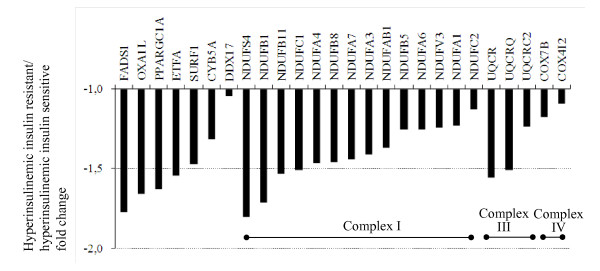
**Mitochondrial respiratory chain pathway was down-regulated in insulin-resistant subjects**. The x-axis shows the fold change of gene expression in the mitochondrial respiratory chain pathway (GO: 0005746) under hyperinsulinemic conditions in the insulin-resistant compared to the insulin-sensitive subjects. Genes encoding mitochondrial complexes I, III and IV are indicated.

Reduced mRNA expression of mitochondrial pathways was a predominant phenotype in the insulin-resistant group during hyperinsulinemia (Table 5). Especially genes in the mitochondrial respiratory chain (GO: 0005746, p = 9.7E-06) pathway were expressed at markedly reduced levels in the insulin-resistant group as compared with the insulin-sensitive group. The genes were mostly those involved in mitochondrial complex I function (Figure 3). In addition to genes encoding mitochondrial complexes I, III, and IV, the differentially expressed genes in this pathway included fatty acid desaturase 1 (*FADS1*) and peroxisome proliferator-activated receptor gamma, coactivator 1 alpha (*PPARGC1) *transcribed at reduced levels in the insulin-resistant women.

**Table 5:** Ten most down-regulated pathways in insulin-resistant compared to insulin-sensitive group during euglycemic hyperinsulinemic conditions

GO category	Pathway name	Nominal *p*	Permuted *p*
GO:0005746	mitochondrial respiratory chain	9.7E-06	0.0002

GO:0044429	mitochondrial part	1.3E-05	0.001

GO:0022900	electron transport chain	1.6E-05	0.0002

GO:0050890	cognition	2.6E-05	0.0005

GO:0016651	oxidoreductase activity, acting on NADH or NADPH	3.9E-05	0.0002

GO:0005759	mitochondrial matrix	4.9E-05	0.0008

GO:0031349	positive regulation of defense response	7.3E-05	0.0008

GO:0022891	substrate-specific transmembrane transporter activity	8.9E-05	0.0022

GO:0003954	NADH dehydrogenase activity	1.0E-04	0.0023

GO:0055114	oxidation reduction	1.1E-04	0.0037

### Induction of lipid metabolism pathways by insulin in the two study groups

**Figure 4:**
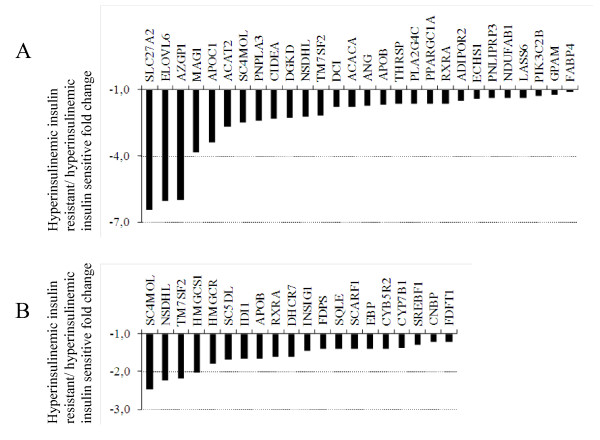
**Lipid and sterol metabolic process pathways were down-regulated in insulin-resistant subjects**. The x-axis shows the fold change of gene expression in the lipid metabolic process (GO:0006629) (A) and sterol metabolic process (GO:0016125) (B) pathways under hyperinsulinemic conditions in the insulin-resistant compared to the insulin-sensitive subjects.

We next investigated the pathways most prominently induced by insulin (Tables 6 &7). The ten most up-regulated pathways were predominantly those involved in lipid metabolism, in both the insulin-resistant and the insulin-sensitive groups. More detailed analysis revealed that the lipid metabolic process (GO:0006629) and sterol metabolic process (GO:0016125) pathways, despite that fact that they were induced by insulin in both groups, were expressed at lower levels in the insulin-resistant as compared with the insulin-sensitive group during hyperinsulinemia (Figure 4A &4B). Fatty acid binding protein 2 (*FATP2 *or *SLC27A2*), fatty acid elongation factor 6 (*ELOVL6*), and patatin-like phospholipase domain containing 3 *(PNPLA3) *were among the most divergently expressed genes in the GO:0006629 pathway. The sterol metabolic process pathway contained several genes with well established roles in cholesterol metabolism, including 3-hydroxy-3-methylglutaryl-Coenzyme A reductase (*HMGCR*) and sterol regulatory element binding transcription factor 1 (*SREBF1*) (Figure 4B).

**Table 6:** Ten most up-regulated pathways by insulin in the insulin-resistant group

GO category	Pathway name	Nominal *p*	Permuted *p*
GO:0016125	sterol metabolic process	1.52E-08	0.0001

GO:0006629	lipid metabolic process	2.31E-08	0.0001

GO:0008203	cholesterol metabolic process	1.12E-07	0.0001

GO:0044255	cellular lipid metabolic process	1.36E-07	0.0001

GO:0008202	steroid metabolic process	6.34E-07	0.0001

GO:0016126	sterol biosynthetic process	4.45E-06	0.0002

GO:0006694	steroid biosynthetic process	4.93E-06	0.0001

GO:0001889	liver development	6.62E-06	0.0002

GO:0008015	blood circulation	1.00E-05	0.0002

GO:0042127	regulation of cell proliferation	1.77E-05	0.0006

**Table 7:** Ten most up-regulated pathways by insulin in the insulin-sensitive group

GO category	Pathway name	Nominal *p*	Permuted *p*
GO:0048534/GO:0002520	hemopoietic or lymphoid organ development/immune system development	1.43E-05	0.0004

GO:0016125	sterol metabolic process	1.94E-05	0.0005

GO:0006469	negative regulation of protein kinase activity	2.86E-05	0.0002

GO:0009725	response to hormone stimulus	3.06E-05	0.001

GO:0030684	Preribosome	3.95E-05	0.0002

GO:0051348	negative regulation of transferase activity	4.78E-05	0.0003

GO:0006629	lipid metabolic process	5.94E-05	0.0023

GO:0008203	cholesterol metabolic process	6.44E-05	0.001

GO:0009887	organ morphogenesis	7.59E-05	0.0029

GO:0030097	hemopoiesis	7.68E-05	0.0023

ANOVA analysis of single insulin-regulated genes revealed a number of additional interesting lipid metabolism-related transcripts whose levels differed between the insulin-sensitive and -resistant groups (Additional file 4: Table S3^Q4^). One of these, angiopoietin-like protein 4 (*ANGPTL4 *or *FIAF*), is currently recognized as an important regulator of lipoprotein lipase function, the expression of which is strongly induced in mouse adipose tissue during fasting [23]. This transcript was elevated during hyperinsulinemia in the insulin-resistant as compared to the insulin-sensitive group.

### Quantitative RT-PCR analysis of mRNA levels of selected genes altered by insulin or insulin resistance status

**Figure 5:**
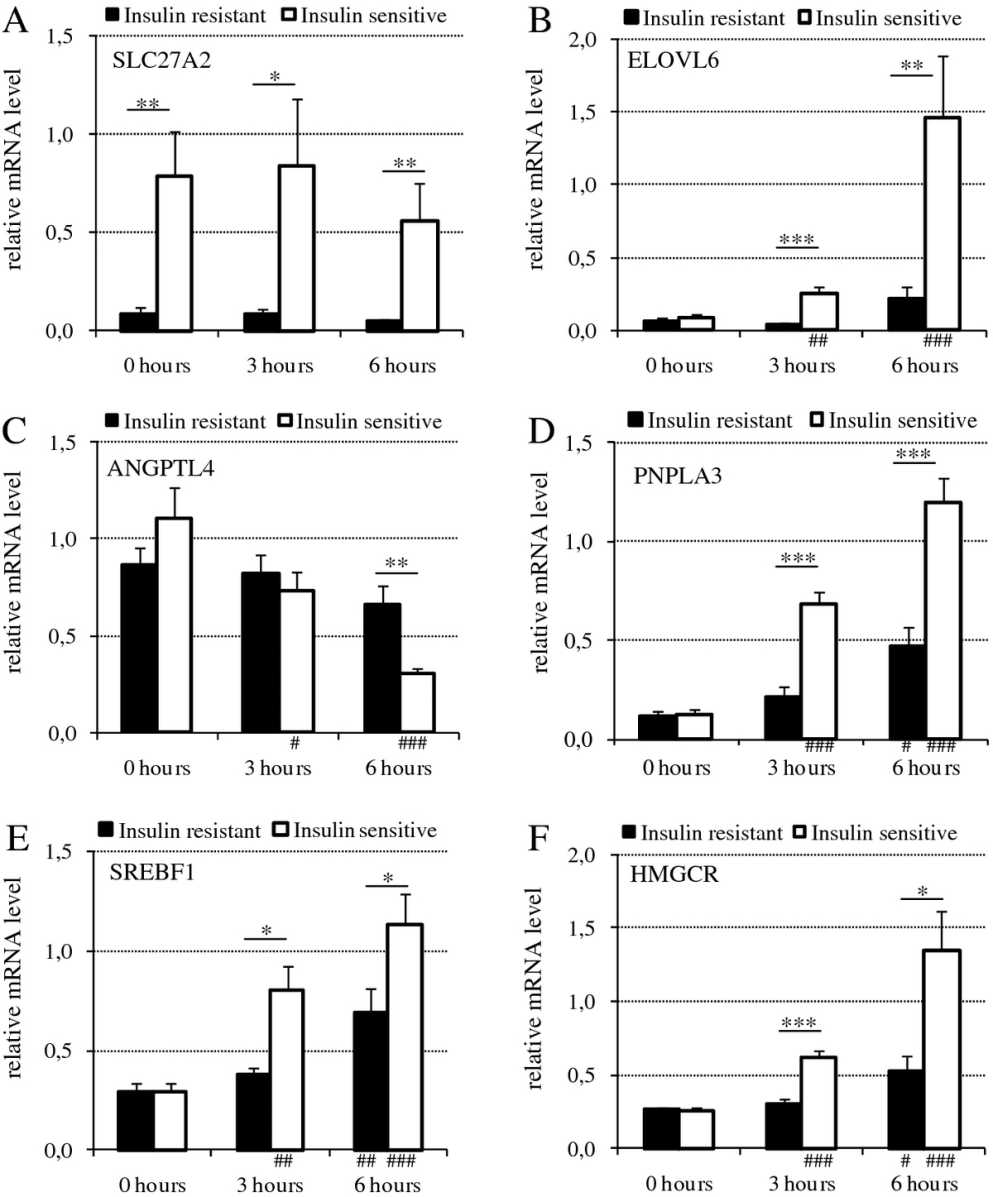
**Relative mRNA levels of genes involved in lipid metabolism and inflammation**. Expression levels were analyzed in the insulin-resistant (black bars) and the insulin-sensitive subjects (white bars) at 0 h, 3 h, and 6 h during euglycemic hyperinsulinemia. *p < 0.05, **p < 0.01, ***p < 0.001 for change between insulin-resistant and insulin-sensitive groups at each time point; #p < 0.05, ##p < 0.01, ###p < 0.001 for change in response to euglycemic hyperinsulinemia between time points at 0 h vs. 3 h and 0 h vs. 6 h.

To verify some of the most interesting findings of the microarray study, we performed qPCR quantification of mRNAs for 8 genes, for 11 insulin-sensitive and 9 insulin-resistant women, under fasting and at the 3 h and 6 h time points of hyperinsulinemia. In all cases, the microarray findings were confirmed. *FATP2 *mRNA remained at lower levels in the insulin-resistant group during fasting (p < 0.01) and the levels were not affected by hyperinsulinemia in either group (Figure 5A). *ELOVL6 *was strongly induced by insulin only in the insulin-sensitive group (p < 0.001) resulting in strikingly decreased mRNA levels in the insulin-resistant compared to insulin-sensitive group (p < 0.01) at the 6 hour time point (Figure 5B). *ANGPTL4 *was strongly down-regulated by insulin in the insulin-sensitive group (p = < 0.001) whereas this regulation was almost absent in the insulin-resistant group (p = NS). As a consequence we observed higher *ANGPTL4 *expression in the insulin-resistant compared to the insulin-sensitive group after 6 hours of hyperinsulinemia (Figure 5C). Furthermore, significantly higher mRNA levels of *ANGPTL4 *in the insulin-sensitive group were observed during fasting in analysis of the 5 most insulin-resistant and 5 most insulin-sensitive persons analyzed in the microarray experiment (p < 0.05, data not shown). *PNPLA3 *expression was lower in the insulin-resistant compared to the insulin-sensitive group during hyperinsulinemia (p < 0.001 at 3 h and p < 0.001 at 6 h) and the expression was increased by insulin in both groups at 6 h time point and in the insulin-sensitive group already at 3 h time point (p < 0.001) (Figure 5D). Expression of *SREBF1 *and its target *HMGCR *displayed significantly lower mRNA levels in the insulin-resistant group after 3 h (p < 0.05 and p < 0.001) and 6 h (p < 0.05 for both genes) of hyperinsulinemia (Figure 5E &5F). The expression of both *SREBF1 *(p < 0.001 at 6 h for insulin-sensitive, p < 0.01 for insulin-resistant) and *HMGCR *(p < 0.001 at 6 h for the insulin-sensitive, p < 0.05 for the insulin-resistant) in both study groups was up-regulated by insulin, although the activation was clearly weaker in the insulin-resistant group. Expression of three nuclear mitochondrial genes, NDUFS4, UQCR and COX4I2, was verified by RT-PCR (Additional file 5). All have lower expression in insulin-resistant as compared to insulin-sensitive subjects, consistently at every time point (p < 0.05).

## Discussion

In the present study we compared the acute *in vivo *effects of insulin on adipose tissue transcriptional profiles of obese insulin-resistant and lean insulin-sensitive women, to gain insight into the molecular mechanisms underlying insulin resistance. The most striking difference in gene expression of the insulin-resistant group during hyperinsulinemia was reduced transcription of genes involved in mitochondrial respiration (mitochondrial respiratory chain, GO:0001934). Inflammatory pathways with complement components (inflammatory response, GO:0006954) and cytokines (chemotaxis, GO:0042330) were strongly up-regulated in insulin-resistant as compared to insulin-sensitive subjects both before and during hyperinsulinemia. Furthermore, differences were observed in genes contributing to fatty acid, cholesterol and triglyceride metabolism and in genes involved in regulating lipolysis, between the insulin-resistant and -sensitive subjects especially during hyperinsulinemia.

Low-grade adipose tissue inflammation has been postulated as one of the central factors in the development of insulin resistance. Increased inflammation was a predominant phenotype in the insulin-resistant vs. insulin-sensitive subjects in the present study. Many findings of the up-regulated genes in the insulin-resistant group, such as *C1Q *peptides, *MMP9 *and *SPP1*, are consistent with previous reports on adipose tissue gene expression in the transcriptomics analysis in the fasting state in obese mice [24,25], and in monozygotic twins discordant for BMI [12]. While these data characterize changes in adipose tissue transcriptome in humans and are unique as such, the role of complement components and inflammatory mediators in the development of insulin resistance cannot be determined in a cross-sectional study in humans. Since many of the inflammatory genes observed in this study are active in cells of the monocyte/macrophage lineage, our observation may reflect increased inflammatory cell content in adipose tissue of the insulin-resistant women. This is in line with the observation that the adipose tissue of lean subjects usually consists of approximately 5-10% of macrophages, whereas in obese patients, adipose tissue macrophage content can be as high as 50% of the total cell number [26]. However, there is increasing evidence that some of the genes we found up-regulated are expressed also by adipocytes, such as complement components, *PLA2G7 *[27,28], *SYK *[29] and *ITGB2*[28]. Adipocyte and macrophage trancriptomes are similar and they can become even more similar after macrophages engulf lipids [15] from the dying adipocytes observed in obesity [30].

Alterations in fatty acid handling and release characterize adipose tissue in obesity. In the present study one of the most notable changes in adipose tissue transcriptome of the insulin-resistant women during fasting was down-regulation of the monocarboxylic acid metabolism pathway, *FATP2 *being the most down-regulated gene in this pathway. There are no previous reports describing *FATP2 *expression in human adipose tissue. Moreover, *FATP2 *is not expressed in mouse 3T3-L1 adipocytes [31] and in murine tissues it is expressed most strongly in liver and kidney cortex (data on adipose tissue not available in this study) [32]. It seems unlikely that the *FATP2 *signal in our study would originate from macrophages, since its transcription is strongly down-regulated in the obese insulin-resistant group where macrophage numbers are elevated [26]. Interestingly, a recent report in rat peripheral blood mononuclear cells suggested *FATP2 *to be an early marker of obesity [33].

*ELOVL6 *is a fatty acid elongation factor specific for long chain fatty acids [34]. It which was recently shown to be regulated by SREBP1c [35]. In the present study we made a novel observation of *ELOVL6 *down-regulation in the obese insulin resistant as compared to the lean insulin-sensitive group in hyperinsulinemia. Interestingly, liver deficiency of *ELOVL6 *significantly ameliorates insulin resistance in mice by modifying hepatic fatty acid composition [36]. PNPLA3 is expressed mainly in liver and adipose tissue and its genetic variants associate in multiple studies with increased hepatic fat. PNPLA3 has been shown to have a lipolytic and weak lipogenic effect in vivo but its precise role in vivo is unclear [37]. Its expression in adipose tissue has been reported to be similar [38,39] or increased [40] in obese compared to lean patients at fasting state. Both insulin and glucose stimulate adipose tissue PNPLA3 expression [39]. The dramatically lower transcript levels of *ELOVL6 *as well as *PNPLA3 *in hyperinsulinemic insulin-resistant subjects might have an impact to adipocyte lipid composition and could further decline adipose tissue function in insulin resistance.

Mitochondrial pathways, especially genes involved in mitochondrial respiration, have been shown to be down-regulated in muscle and adipose tissues of insulin-resistant and type 2 diabetic subjects in fasting state [41,42]. Acquired obesity and poor physical fitness are known to impair the expression of genes of oxidative phosphorylation (41). However, their response to insulin in insulin resistant subjects has not been reported before. Mootha et al. (41) presented evidence that a number of genes involved in oxidative phosphorylation in skeletal muscle are subject to regulation by PGC-1α encoded by *PPARGC1*, and are down-regulated in type 2 diabetes. Therefore, the down-regulation of *PPARGC1 *we observed in the subcutaneous fat of insulin resistant subjects may provide one mechanistic explanation to the reduced expression of mitochondrial respiratory chain genes. Interestingly, decreased expression of mitochondrial pathways is the most prominent finding during hyperinsulinemia reflecting a regulatory defect that may further aggravate the pathogenesis of insulin resistance. Since regular practice of physical exercise is known to improve insulin sensitivity and reduce body weight [43,44], it is possible that differences in physical activity could have amplified the observed differences in gene expression between the insulin sensitive and insulin resistant subjects.

Activity of lipoprotein lipase (LPL) is an important determinant in the development of obesity in mouse models. As a general rule, high fat diet-induced adipogenesis is aggravated by stimulated LPL activity (e.g. by adipose tissue-specific over expression of *LPL *or deficiency for APOCIII), and attenuated by inhibited LPL activity [45]. A physiologically important LPL inhibitor, ANGPTL4, is strongly down-regulated by insulin in mouse 3T3-L1 cells [46]. In the present study we found insulin to significantly decrease *ANGPTL4 *expression in human adipose tissue. This finding is novel as was the finding of impaired acute insulin regulation of *ANGPTL4 *in obese insulin resistant as compared to lean insulin sensitive subjects. In mice, *ANGPTL4 *over expression inhibits LPL, which in turn slows down adipogenesis and increases plasma triglyceride concentrations. When *ANGPTL4 *is deleted the reverse phenotype arises [23,45,47]. Interestingly, serum ANGPTL4 levels are inversely correlated with plasma glucose concentrations and the serum levels are significantly lower in type 2 diabetic patients than healthy subjects [48]. ANGPTL4 expression is also stimulated by insulin sensitizing drugs thiazolidinediones via PPARy mediated mechanism suggesting that changes in ANGPTL level could have a role in the development of insulin-resistance [49].

The insulin-resistant group displayed defective induction of important insulin-induced lipid metabolism pathways. While several genes in these pathways are important players in cholesterol metabolism and have a well established role in cholesterol synthesis in liver, little is known of their importance in adipose tissue. Adipose tissue contains the body's largest pool of free cholesterol and cholesterol imbalance is recognized as a characteristic of enlarged adipocytes in obesity [50]. Therefore, the differentially expressed genes in the sterol metabolic pathway in our data could play an important part in the insulin-resistant phenotype. The molecular mechanism underlying the decreased expression of lipid biosynthesis pathways in the insulin-resistant group could involve SREBP-1c since several of the genes shown in Figure 4 are targets of SREBP-1c including *HMGCR *and *ELOVL6 *[35,51]. SREBP-1c is regulated mainly at the transcriptional level and is a major mediator of fatty acid and triglyceride synthesis induction by insulin in liver and adipose tissue [51-54]. Down-regulation of SREBP-1c was observed in the adipose tissue of leptin deficient mice (ob/ob) [55], in insulin-resistant humans during fasting and in response to insulin [56], and in adipose tissue of patients with type 2 diabetes [57], similar to our study. In contrast to the latter study, we detected no difference in *SREBF1 *expression between the groups in the basal state. The small sample size of the study could have contributed to failure to detect such a difference and also other small scale changes in gene expression. The defective response of *SREBF1 *to insulin in the insulin-resistant group could explain down-regulation of *ELOVL6 *and *HMGCR *mRNAs in this group. Reduced *SREBF1 *transcription could be a homeostatic reaction of enlarged adipocytes to prevent further lipid synthesis. On the other hand, the reduced *SREBF1 *expression could also decrease lipogenesis in smaller adipocytes and hinder adipocyte differentiation via *PPARG *down-regulation, leading to increased insulin resistance [58].

## Conclusions

In conclusion, our data demonstrate that insulin-resistant subjects have a marked decrease in the expression of numerous genes involved in lipid and mitochondrial metabolism in human adipose tissue. This study also replicates previous findings of increased inflammation in adipose tissue of insulin-resistant human subjects. Insulin resistance is a complex phenotype characterized by disturbances in the transcriptional networks controlling immune response, lipid metabolism and mitochondrial function. While this study uncovers several novel aberrations in the obese and insulin-resistant human subjects' adipose tissue, the ultimate trigger of the transcriptional changes leading to adipose tissue dysfunction and insulin resistance remains to be identified.

## Competing interests

The authors declare that they have no competing interests.

## Authors' contributions

JS researched data and drafted the manuscript. PPL, JN, IS and SR researched data and edited the manuscript and contributed to discussion. MJ and VMO edited the manuscript and contributed to discussion. HJ designed the clinical study, edited the manuscript and contributed to discussion. All authors read and approved the final manuscript.

## Pre-publication history

The pre-publication history for this paper can be accessed here:

http://www.biomedcentral.com/1755-8794/5/9/prepub

## Supplementary Material

Additional file 1 **R Statistics code for Mixed Effects ANOVA**. Click here for file

Additional file 2 **Primer sequences**. Click here for file

Additional file 3 **2-way ANOVA of differentially expressed genes associated with insulin resistance status**. All probes with p-value < 0.05 after Benjamini-Hochberg correction are reported in ascending order. Non-corrected *p*-values are reported. Fold change (FC) and direction of regulation in insulin-resistant compared to insulin-sensitive group are reported in fasting state and during hyperinsulinemia. Click here for file

Additional file 4 **2-way ANOVA of differentially expressed genes associated with hyperinsulinemia**. All probes with p-value < 0.05 after Benjamini-Hochberg correction are reported in ascending order. Non-corrected p-values are reported. Fold change (FC) and direction of regulation in insulin-resistant compared to insulin-sensitive group are reported in fasting state and during hyperinsulinemia. Click here for file

Additional file 5 **Relative mRNA levels of selected genes in mitochondrial respiratory chain pathway**. Expression levels were analyzed in insulin-resistant (black bar) and insulin-sensitive subjects (white bar) at 0 h, 3 h, and 6 h during euglycaemic hyperinsulinaemia. All genes are differentially expressed between insulin-resistant and insulin-sensitive groups: p(group) < 0.05, p(group*insulin) = NS, p(insulin) = NS for all genes via 2-way ANOVA. Click here for file
