# Supplementary material for: Adipose tissue gene expression analysis reveals changes in inflammatory, mitochondrial respiratory and lipid metabolic pathways in obese insulin-resistant subjects
Source: BMC Med Genomics. 2012 Apr 3;5:9. doi: 10.1186/1755-8794-5-9 (PMC3384471; doi:10.1186/1755-8794-5-9)
Supplement: Additional file 1 — R Statistics code for Mixed Effects ANOVA. [file 1755-8794-5-9-S1.DOC]

**Additional file 1**

**R Statistics code for Mixed Effects ANOVA**

#

a<-read.table("sd-filter_formixedmodelANOVAwithIda.tsv", header=T, as.is=T, sep="\t")

a<-a[,1:22]

out<-as.data.frame(a[,1])

out$P_value_Treatment<-rep(NA)

out$P_value_Case<-rep(NA)

out$P_value_inter<-rep(NA)

idt<-read.table("phenotypes.txt", as.is=T, sep="\t")

m<-match(names(a[,3:length(a[1,])]),idt[,1])

idt

for(i in 1:length(a[,1])){

tmp<-a[i,]

tmp2<-t(tmp)

tmp2<-tmp2[3:length(tmp2[,1]),]

tmp2<-as.data.frame(tmp2)

names(tmp2)<-"Expression"

tmp2$ID<-c(seq(1,10),seq(1,10))

tmp2$Case<-rep(c(1,0,1,0),each=5)

tmp2$Treatment<-rep(c(1,0),each=10)

tmp2$Expression<-as.numeric(as.character(tmp2$Expression))

#fit<-aov(Expression~Case+Error(ID / Treatment),data=tmp2)

#fit<-aov(Expression~Treatment+Error(Case / ID),data=tmp2)

#fit<-aov(Expression~Treatment+Error(ID),data=tmp2)

fit<-aov(Expression~(as.factor(Case)*as.factor(Treatment))+Error(as.factor(ID)/(as.factor(Treatment)))+as.factor(Case),data=tmp2)

out$P_value_Case[i]<-unlist(summary(fit)$"Error: as.factor(ID)")[9]

out$P_value_Treatment[i]<-unlist(summary(fit)$"Error: as.factor(ID):as.factor(Treatment)")[13]

out$P_value_inter[i]<-unlist(summary(fit)$"Error: as.factor(ID):as.factor(Treatment)")[14]

}

head(out)

### P-value: Benjamini-Hochberg correction for multiple testing

out<-out[order(out$P_value_Treatment),]

out$Treatment_corrected<-rep(NA)

out$rank_tmp<-seq(1,length(out[,1]))

out<-out[order(out$P_value_Treatment,decreasing=T),]

out$Treatment_corrected<-(length(out[,1])/out$rank_tmp)*out$P_value_Treatment

out<-out[order(out$P_value_Case),]

out$Case_corrected<-rep(NA)

out$rank_tmp<-seq(1,length(out[,1]))

out<-out[order(out$P_value_Case,decreasing=T),]

out$Case_corrected<-(length(out[,1])/out$rank_tmp)*out$P_value_Case

out<-out[order(out$P_value_inter),]

out$inter_corrected<-rep(NA)

out$rank_tmp<-seq(1,length(out[,1]))

out<-out[order(out$P_value_inter,decreasing=T),]

out$inter_corrected<-(length(out[,1])/out$rank_tmp)*out$P_value_inter

m<-match(out[,1],a$symbol)

out<-cbind(out,a[m,3:length(a[1,])])

out$at_symbol<-row.names(a[m,])

write.table(out[,-6],"Results_file.txt", row.names=F, quote=F, sep="\t")
