# Supplementary material for: Adipose tissue gene expression analysis reveals changes in inflammatory, mitochondrial respiratory and lipid metabolic pathways in obese insulin-resistant subjects
Source: BMC Med Genomics. 2012 Apr 3;5:9. doi: 10.1186/1755-8794-5-9 (PMC3384471; doi:10.1186/1755-8794-5-9)
Supplement: Additional file 2 — Primer sequences. [file 1755-8794-5-9-S2.DOC]

**Additional file 2**

**Primer sequences.**

| **Gene** | **Forward primer** | **Reverse Primer** |
| --- | --- | --- |
| FATP2 | GTGGAGAAAGATGAACCTGTCCG | CTGAGCCTTTGCTCCAGCATAG |
| ELOVL6 | CCATCCAATGGATGCAGGAAAAC | CCAGAGCACTAATGGCTTCCTC |
| APOC1 | AGGACAAGGCTCGGGAACTCAT | GATGTCACCCTTCAGGTCCTCA |
| SREBF1 | ACTTCTGGAGGCATCGCAAGCA | AGGTTCCAGAGGAGGCTACAAG |
| HMGCR | GACGTGAACCTATGCTGGTCAG | GGTATCTGTTTCAGCCACTAAGG |
| ANGPTL4 | GATGGCTCAGTGGACTTCAACC | TGCTATGCACCTTCTCCAGACC |
| ACTB | CACCATTGGCAATGAGCGGTTC | AGGTCTTTGCGGATGTCCACGT |
| RPLP0 | TGGTCATCCAGCAGGTGTTCGA | ACAGACACTGGCAACATTGCGG |
| NDUFS4 | GAGATTGGCACAGGACCAGACT | CCAGACTGCATGTTATTGCGAGC |
| UQCR11 | CTGGTGTGGGCCACCGATTGG | CAGAGCAGTCTGTGAAGGGTTTG |
| COX4I2 | CCTTCTGCACAGAACTCAACGC | AGGTCTCATTGAACTGGAGCCG |
