# Supplementary material for: Adipose tissue gene expression analysis reveals changes in inflammatory, mitochondrial respiratory and lipid metabolic pathways in obese insulin-resistant subjects
Source: BMC Med Genomics. 2012 Apr 3;5:9. doi: 10.1186/1755-8794-5-9 (PMC3384471; doi:10.1186/1755-8794-5-9)
Supplement: Additional file 3 — 2-way ANOVA of differentially expressed genes associated with insulin resistance status. All probes with p-value < 0.05 after Benjamini-Hochberg correction are reported in ascending order. Non-corrected p-values are reported. Fold change (FC) and direction of regulation in insulin-resistant compared to insulin-sensitive group are reported in fasting state and during hyperinsulinemia. [file 1755-8794-5-9-S3.DOC]

**Additional file 3.**

**2-way ANOVA of differentially expressed genes associated with insulin resistance status.**

All probes with p-value <0.05 after Benjamini-Hochberg correction are reported in ascending order. Non-corrected *p*-values are reported. Fold change (FC) and direction of regulation in insulin-resistant compared to insulin-sensitive group are reported in fasting state and during hyperinsulinemia.

|  | | | **genes regulated by resistance status** | | | **Hyperinsuliaemic insulin-resistant/ hyperinsulinaemic insulin-sensitive** | | **fasted insulin-resistant/ fasted insulin-sensitive** | |
| --- | --- | --- | --- | --- | --- | --- | --- | --- | --- |
|  | **gene symbol** | **probe** | ***p* group** | ***p* insulin** | ***p* Interaction** | **FC** | **regulation** | **FC** | **regulation** |
| 1 | GLB1 | 2720_at | 2,6E-05 | NS | NS | 4,62 | up | 3,09 | up |
| 2 | FUCA1 | 2517_at | 4,9E-05 | NS | 0,043 | 3,99 | up | 2,39 | up |
| 3 | GPR137B | 7107_at | 5,1E-05 | NS | NS | 7,49 | up | 7,53 | up |
| 4 | C12orf5 | 57103_at | 7,0E-05 | NS | NS | 2,58 | up | 3,83 | up |
| 5 | PLA2G7 | 7941_at | 8,1E-05 | NS | NS | 20,70 | up | 24,00 | up |
| 6 | SFRP2 | 6423_at | 9,1E-05 | NS | NS | 8,29 | up | 6,25 | up |
| 7 | SLC38A6 | 145389_at | 1,2E-04 | NS | NS | 3,66 | up | 2,65 | up |
| 8 | CD52 | 1043_at | 1,8E-04 | NS | NS | 5,91 | up | 7,66 | up |
| 9 | NCEH1 | 57552_at | 1,9E-04 | NS | NS | 8,30 | up | 12,67 | up |
| 10 | CFB | 629_at | 1,9E-04 | NS | NS | 2,63 | up | 2,55 | up |
| 11 | FAM105A | 54491_at | 2,1E-04 | NS | NS | 3,06 | up | 3,47 | up |
| 12 | DOCK2 | 1794_at | 3,4E-04 | NS | NS | 4,97 | up | 4,02 | up |
| 13 | CTSG | 1511_at | 3,4E-04 | NS | NS | 2,02 | up | 4,41 | up |
| 14 | LPXN | 9404_at | 3,5E-04 | NS | NS | 3,99 | up | 3,55 | up |
| 15 | PALLD | 23022_at | 3,6E-04 | NS | NS | 2,95 | up | 3,28 | up |
| 16 | FRMD4B | 23150_at | 4,0E-04 | NS | NS | 3,63 | up | 3,67 | up |
| 17 | SLC27A2 | 11001_at | 4,2E-04 | NS | NS | 6,44 | down | 9,14 | down |
| 18 | ALCAM | 214_at | 4,6E-04 | NS | NS | 8,63 | up | 6,13 | up |
| 19 | SYK | 6850_at | 4,7E-04 | NS | NS | 3,83 | up | 7,52 | up |
| 20 | LAPTM5 | 7805_at | 5,0E-04 | NS | NS | 4,45 | up | 4,55 | up |
| 21 | NCF2 | 4688_at | 5,6E-04 | NS | NS | 7,98 | up | 6,17 | up |
| 22 | FCGR2B | 2213_at | 6,0E-04 | NS | NS | 9,03 | up | 7,98 | up |
| 23 | HCLS1 | 3059_at | 6,2E-04 | NS | NS | 2,63 | up | 2,76 | up |
| 24 | IGSF6 | 10261_at | 6,2E-04 | NS | NS | 11,30 | up | 5,42 | up |
| 25 | KIT | 3815_at | 6,3E-04 | NS | NS | 3,81 | up | 2,71 | up |
| 26 | ITGB2 | 3689_at | 6,5E-04 | NS | NS | 6,02 | up | 6,44 | up |
| 27 | LYZ | 4069_at | 6,6E-04 | NS | NS | 6,69 | up | 10,86 | up |
| 28 | MS4A4A | 51338_at | 7,1E-04 | NS | NS | 6,92 | up | 5,78 | up |
| 29 | CTSS | 1520_at | 7,2E-04 | NS | NS | 6,86 | up | 6,36 | up |
| 30 | NPL | 80896_at | 7,5E-04 | NS | NS | 7,84 | up | 10,72 | up |
| 31 | RGS10 | 6001_at | 8,1E-04 | NS | NS | 3,64 | up | 4,01 | up |
| 32 | SCIN | 85477_at | 8,1E-04 | NS | NS | 2,62 | up | 3,86 | up |
| 33 | F13A1 | 2162_at | 8,8E-04 | NS | NS | 3,38 | up | 4,47 | up |
| 34 | RNASE6 | 6039_at | 8,9E-04 | NS | NS | 3,24 | up | 5,70 | up |
| 35 | LOC644285 | 644285_at | 9,6E-04 | NS | NS | 1,76 | down | 3,12 | down |
| 36 | CPA3 | 1359_at | 9,6E-04 | NS | NS | 5,60 | up | 4,39 | up |
| 37 | COL6A6 | 131873_at | 9,6E-04 | NS | NS | 3,95 | down | 5,17 | down |
| 38 | SPP1 | 6696_at | 9,7E-04 | NS | NS | 19,58 | up | 27,69 | up |
| 39 | TYROBP | 7305_at | 9,9E-04 | NS | NS | 3,72 | up | 7,36 | up |
| 40 | SLC7A7 | 9056_at | 9,9E-04 | NS | NS | 4,96 | up | 4,68 | up |
| 41 | C1QA | 712_at | 1,0E-03 | NS | NS | 5,12 | up | 4,08 | up |
| 42 | ATP1B1 | 481_at | 1,0E-03 | NS | NS | 5,06 | up | 4,27 | up |
| 43 | CKAP2 | 26586_at | 1,2E-03 | NS | NS | 3,39 | up | 1,44 | up |
| 44 | C1orf162 | 128346_at | 1,2E-03 | NS | NS | 3,32 | up | 4,60 | up |
| 45 | NIPSNAP3B | 55335_at | 1,3E-03 | NS | NS | 2,56 | down | 3,46 | down |
| 46 | C6orf192 | 116843_at | 1,3E-03 | NS | NS | 3,23 | up | 2,39 | up |
| 47 | TNFSF13B | 10673_at | 1,4E-03 | NS | NS | 2,86 | up | 2,68 | up |
| 48 | PFKFB3 | 5209_at | 1,4E-03 | 3,9E-06 | NS | 1,88 | down | 1,65 | down |
| 49 | C1QB | 713_at | 1,4E-03 | NS | NS | 9,13 | up | 9,67 | up |
| 50 | CD53 | 963_at | 1,4E-03 | NS | NS | 5,02 | up | 4,57 | up |
| 51 | NRIP3 | 56675_at | 1,5E-03 | NS | NS | 3,16 | up | 3,41 | up |
| 52 | SLC39A11 | 201266_at | 1,6E-03 | NS | NS | 2,81 | up | 1,98 | up |
| 53 | GLIPR1 | 11010_at | 1,6E-03 | NS | NS | 5,13 | up | 4,92 | up |
| 54 | MXRA5 | 25878_at | 1,6E-03 | NS | NS | 4,69 | up | 4,01 | up |
| 55 | BLNK | 29760_at | 1,6E-03 | NS | NS | 4,89 | up | 3,56 | up |
| 56 | NCF4 | 4689_at | 1,6E-03 | NS | NS | 2,33 | up | 3,43 | up |
| 57 | TMEM176B | 28959_at | 1,7E-03 | NS | NS | 3,22 | up | 2,43 | up |
| 58 | CLIC6 | 54102_at | 1,7E-03 | NS | NS | 6,27 | up | 4,34 | up |
| 59 | DYRK2 | 8445_at | 1,7E-03 | NS | NS | 3,43 | up | 1,74 | up |
| 60 | SLIT2 | 9353_at | 1,7E-03 | NS | NS | 2,93 | up | 2,41 | up |
| 61 | SFRP4 | 6424_at | 1,8E-03 | NS | NS | 9,52 | up | 3,59 | up |
| 62 | BMP2 | 650_at | 1,8E-03 | 2,0E-03 | 0,014 | 5,69 | up | 1,97 | up |
| 63 | ITGAM | 3684_at | 2,0E-03 | NS | NS | 4,97 | up | 3,77 | up |
| 64 | COTL1 | 23406_at | 2,0E-03 | NS | NS | 3,68 | up | 3,50 | up |
| 65 | CTSZ | 1522_at | 2,0E-03 | NS | NS | 2,28 | up | 2,33 | up |
| 66 | EPB41L3 | 23136_at | 2,0E-03 | NS | 0,034 | 3,42 | up | 1,85 | up |
| 67 | CDKN1A | 1026_at | 2,2E-03 | 6,4E-05 | 4,8E-03 | 3,49 | down | 1,20 | down |
| 68 | MS4A6A | 64231_at | 2,3E-03 | NS | NS | 4,82 | up | 4,76 | up |
| 69 | NEXN | 91624_at | 2,3E-03 | 0,034 | NS | 4,21 | up | 1,82 | up |
| 70 | SLC2A10 | 81031_at | 2,4E-03 | NS | NS | 2,79 | up | 1,81 | up |
| 71 | MS4A7 | 58475_at | 2,4E-03 | 0,044 | NS | 2,21 | up | 3,41 | up |
| 72 | HMOX1 | 3162_at | 2,4E-03 | 2,0E-03 | NS | 2,47 | up | 2,56 | up |
| 73 | TNMD | 64102_at | 2,4E-03 | NS | NS | 4,50 | up | 4,66 | up |
| 74 | ODZ4 | 26011_at | 2,4E-03 | NS | NS | 3,40 | up | 2,71 | up |
| 75 | GZMA | 3001_at | 2,5E-03 | NS | NS | 2,59 | up | 2,58 | up |
| 76 | CTSB | 1508_at | 2,5E-03 | NS | NS | 2,68 | up | 2,58 | up |
| 77 | FBP1 | 2203_at | 2,5E-03 | NS | NS | 7,27 | up | 3,33 | up |
| 78 | ARHGEF16 | 27237_at | 2,5E-03 | 2,7E-05 | 1,1E-03 | 2,79 | down | 1,16 | down |
| 79 | AIF1 | 199_at | 2,5E-03 | NS | NS | 2,43 | up | 3,71 | up |
| 80 | MARCO | 8685_at | 2,6E-03 | NS | NS | 8,29 | up | 6,46 | up |
| 81 | PTPRE | 5791_at | 2,6E-03 | NS | NS | 3,10 | up | 3,40 | up |
| 82 | MPEG1 | 219972_at | 2,6E-03 | NS | NS | 4,11 | up | 3,36 | up |
| 83 | PPP1R3B | 79660_at | 2,7E-03 | 5,3E-04 | 0,013 | 4,76 | down | 1,07 | down |
| 84 | SLC2A3P1 | 100128062_at | 2,7E-03 | 1,6E-04 | 4,9E-03 | 2,38 | down | 1,18 | up |
| 85 | TM6SF1 | 53346_at | 2,8E-03 | NS | NS | 3,38 | up | 3,56 | up |
| 86 | VSIG4 | 11326_at | 2,8E-03 | NS | NS | 5,83 | up | 7,35 | up |
| 87 | IQGAP2 | 10788_at | 2,9E-03 | NS | NS | 3,75 | up | 3,50 | up |
| 88 | EGR2 | 1959_at | 3,0E-03 | NS | NS | 8,68 | up | 9,21 | up |
| 89 | IL10RA | 3587_at | 3,0E-03 | NS | NS | 3,01 | up | 4,61 | up |
| 90 | ALDH1A3 | 220_at | 3,0E-03 | NS | NS | 4,55 | up | 5,40 | up |
| 91 | ALPK3 | 57538_at | 3,0E-03 | NS | NS | 2,48 | down | 3,81 | down |
| 92 | TUBB2A | 7280_at | 3,1E-03 | 7,2E-03 | NS | 2,03 | up | 2,54 | up |
| 93 | OLFML2B | 25903_at | 3,2E-03 | NS | NS | 2,17 | up | 2,28 | up |
| 94 | HCK | 3055_at | 3,2E-03 | 0,028 | NS | 2,62 | up | 3,34 | up |
| 95 | MMP9 | 4318_at | 3,3E-03 | NS | NS | 15,21 | up | 12,81 | up |
| 96 | RASSF2 | 9770_at | 3,3E-03 | NS | NS | 3,50 | up | 5,21 | up |
| 97 | TNC | 3371_at | 3,5E-03 | NS | NS | 4,30 | up | 5,25 | up |
| 98 | IFI30 | 10437_at | 3,5E-03 | NS | NS | 9,35 | up | 11,06 | up |
| 99 | LCP1 | 3936_at | 3,6E-03 | 0,031 | NS | 6,38 | up | 4,92 | up |
| 100 | FCER1A | 2205_at | 3,6E-03 | NS | NS | 3,26 | up | 2,77 | up |
| 101 | C1QC | 714_at | 3,8E-03 | NS | NS | 3,99 | up | 4,14 | up |
| 102 | SYNC | 81493_at | 3,8E-03 | 0,010 | 0,010 | 7,21 | up | 2,56 | up |
| 103 | EVI2B | 2124_at | 3,9E-03 | NS | NS | 5,29 | up | 4,07 | up |
| 104 | CLEC10A | 10462_at | 3,9E-03 | NS | NS | 1,75 | up | 3,46 | up |
| 105 | BCAT1 | 586_at | 3,9E-03 | NS | NS | 1,97 | up | 3,34 | up |
| 106 | HPGDS | 27306_at | 4,2E-03 | NS | NS | 3,18 | up | 3,50 | up |
| 107 | UCHL1 | 7345_at | 4,2E-03 | NS | NS | 5,37 | up | 10,12 | up |
| 108 | AGT | 183_at | 4,3E-03 | 8,0E-03 | 0,027 | 3,28 | down | 1,52 | down |
| 109 | ANKDD1A | 348094_at | 4,3E-03 | NS | NS | 5,55 | up | 3,26 | up |
| 110 | SLC31A2 | 1318_at | 4,4E-03 | 2,7E-04 | NS | 2,43 | up | 1,83 | up |
| 111 | FOLR2 | 2350_at | 4,6E-03 | NS | NS | 3,04 | up | 4,88 | up |
| 112 | PLEK | 5341_at | 4,7E-03 | NS | NS | 6,25 | up | 3,93 | up |
| 113 | CD163 | 9332_at | 4,9E-03 | NS | NS | 4,77 | up | 5,12 | up |
| 114 | CD48 | 962_at | 5,0E-03 | NS | NS | 1,78 | up | 3,10 | up |
| 115 | SLA | 6503_at | 5,1E-03 | 4,0E-03 | NS | 2,51 | up | 3,20 | up |
| 116 | COL11A1 | 1301_at | 5,2E-03 | NS | NS | 6,15 | up | 9,76 | up |
| 117 | DHRS9 | 10170_at | 5,3E-03 | NS | NS | 6,23 | up | 7,54 | up |
| 118 | CCL13 | 6357_at | 5,3E-03 | NS | NS | 5,17 | up | 6,54 | up |
| 119 | C2orf40 | 84417_at | 5,3E-03 | NS | NS | 3,41 | down | 3,16 | down |
| 120 | AZGP1 | 563_at | 5,4E-03 | NS | NS | 5,98 | down | 8,98 | down |
| 121 | MELK | 9833_at | 5,4E-03 | NS | NS | 2,58 | up | 3,08 | up |
| 122 | MNDA | 4332_at | 5,5E-03 | NS | NS | 3,74 | up | 4,13 | up |
| 123 | BCL2A1 | 597_at | 5,5E-03 | NS | NS | 3,71 | up | 5,90 | up |
| 124 | GPR183 | 1880_at | 5,5E-03 | NS | NS | 7,76 | up | 5,79 | up |
| 125 | LY96 | 23643_at | 5,6E-03 | NS | NS | 2,45 | up | 2,95 | up |
| 126 | EVI2A | 2123_at | 5,6E-03 | NS | NS | 5,38 | up | 3,90 | up |
| 127 | OGN | 4969_at | 5,6E-03 | NS | NS | 2,45 | up | 1,45 | up |
| 128 | CPVL | 54504_at | 5,7E-03 | NS | NS | 3,10 | up | 4,58 | up |
| 129 | FPR3 | 2359_at | 5,8E-03 | NS | NS | 2,27 | up | 4,13 | up |
| 130 | hCG_25653 | 646360_at | 5,9E-03 | NS | NS | 4,35 | up | 4,08 | up |
| 131 | KIAA1598 | 57698_at | 5,9E-03 | NS | NS | 3,02 | up | 2,32 | up |
| 132 | LILRB2 | 10288_at | 6,0E-03 | NS | NS | 2,55 | up | 3,56 | up |
| 133 | C3AR1 | 719_at | 6,0E-03 | NS | NS | 3,74 | up | 5,65 | up |
| 134 | NPR3 | 4883_at | 6,1E-03 | 9,8E-03 | NS | 3,03 | up | 2,65 | up |
| 135 | MMP7 | 4316_at | 6,5E-03 | NS | NS | 6,41 | up | 8,23 | up |
| 136 | BBOX1 | 8424_at | 6,6E-03 | NS | NS | 2,90 | up | 4,85 | up |
| 137 | CD14 | 929_at | 6,8E-03 | NS | NS | 2,63 | up | 3,80 | up |
| 138 | ITIH5 | 80760_at | 7,0E-03 | NS | 5,0E-03 | 3,01 | up | 1,89 | up |
| 139 | ADAM9 | 8754_at | 7,2E-03 | NS | NS | 3,58 | up | 2,05 | up |
| 140 | CYTIP | 9595_at | 7,4E-03 | 0,045 | NS | 3,43 | up | 2,43 | up |
| 141 | MFHAS1 | 9258_at | 7,4E-03 | NS | 0,014 | 3,86 | up | 1,71 | up |
| 142 | C6orf115 | 58527_at | 7,6E-03 | NS | NS | 2,42 | up | 2,54 | up |
| 143 | SAMSN1 | 64092_at | 7,7E-03 | NS | NS | 2,44 | up | 3,15 | up |
| 144 | RAB31 | 11031_at | 7,7E-03 | NS | NS | 2,51 | up | 2,40 | up |
| 145 | GRB14 | 2888_at | 7,8E-03 | NS | NS | 2,09 | up | 2,29 | up |
| 146 | EGFL6 | 25975_at | 8,0E-03 | NS | NS | 5,95 | up | 18,17 | up |
| 147 | TFEC | 22797_at | 8,1E-03 | NS | NS | 2,31 | up | 3,68 | up |
| 148 | LGMN | 5641_at | 8,2E-03 | NS | NS | 2,27 | up | 2,62 | up |
