# Supplementary material for: Adipose tissue gene expression analysis reveals changes in inflammatory, mitochondrial respiratory and lipid metabolic pathways in obese insulin-resistant subjects
Source: BMC Med Genomics. 2012 Apr 3;5:9. doi: 10.1186/1755-8794-5-9 (PMC3384471; doi:10.1186/1755-8794-5-9)
Supplement: Additional file 4 — 2-way ANOVA of differentially expressed genes associated with hyperinsulinemia. All probes with p-value < 0.05 after Benjamini-Hochberg correction are reported in ascending order. Non-corrected p-values are reported. Fold change (FC) and direction of regulation in insulin-resistant compared to insulin-sensitive group are reported in fasting state and during hyperinsulinemia. [file 1755-8794-5-9-S4.DOC]

**Additional file 4**

**2-way ANOVA of differentially expressed genes associated with hyperinsulinemia.**

All probes with *p*-value <0.05 after Benjamini-Hochberg correction are reported in ascending order. Non-corrected *p*-values are reported. Fold change (FC) and direction of regulation in insulin-resistant compared to insulin-sensitive group are reported in fasting state and during hyperinsulinemia.

|  | | | **Genes regulated by insulin** | | | **Hyperinsuliaemic insulin-resistant/ hyperinsulinaemic insulin-sensitive** | | **fasted insulin-resistant/ fasted insulin-sensitive** | |
| --- | --- | --- | --- | --- | --- | --- | --- | --- | --- |
|  | **Gene Symbol** | **probe** | ***p* Insulin** | ***p* group** | ***p* Interaction** | **FC** | **regulation** | **FC** | **regulation** |
| 1 | PDK4 | 5166_at | 1,20E-06 | NS | 0,016 | 1,77 | up | 1,37 | down |
| 2 | HBP1 | 26959_at | 1,96E-06 | NS | 2,6E-03 | 1,73 | up | 1,25 | down |
| 3 | PNPLA3 | 80339_at | 3,02E-06 | 0,048 | NS | 2,39 | down | 1,71 | down |
| 4 | KIAA0040 | 9674_at | 3,22E-06 | NS | 9,3E-03 | 1,77 | down | 1,32 | up |
| 5 | PFKFB3 | 5209_at | 3,91E-06 | 1,4E-03 | NS | 1,88 | down | 1,65 | down |
| 6 | DDIT4 | 54541_at | 7,10E-06 | NS | NS | 1,66 | up | 1,10 | up |
| 7 | NFIL3 | 4783_at | 7,68E-06 | NS | NS | 2,40 | down | 1,48 | down |
| 8 | SREBF1 | 6720_at | 1,69E-05 | NS | NS | 1,31 | down | 1,15 | up |
| 9 | PPIF | 10105_at | 1,75E-05 | NS | 9,1E-04 | 3,07 | down | 1,06 | down |
| 10 | SC4MOL | 6307_at | 1,79E-05 | NS | 0,039 | 2,48 | down | 1,02 | up |
| 11 | IRS2 | 8660_at | 1,80E-05 | NS | NS | 1,07 | down | 1,60 | down |
| 12 | SLC2A5 | 6518_at | 1,93E-05 | NS | 1,6E-03 | 2,88 | down | 1,33 | up |
| 13 | NANOS1 | 340719_at | 2,54E-05 | NS | NS | 1,02 | up | 1,56 | up |
| 14 | ERRFI1 | 54206_at | 2,54E-05 | NS | 0,020 | 1,55 | up | 1,40 | down |
| 15 | PIK3IP1 | 113791_at | 2,58E-05 | NS | NS | 1,61 | up | 1,04 | up |
| 16 | ARHGEF16 | 27237_at | 2,74E-05 | 2,5E-03 | 1,1E-03 | 2,79 | down | 1,16 | down |
| 17 | C7orf68 | 29923_at | 3,11E-05 | NS | 0,027 | 2,44 | up | 1,25 | down |
| 18 | CEBPB | 1051_at | 3,21E-05 | NS | 0,049 | 1,62 | down | 1,09 | down |
| 19 | SPATA7 | 55812_at | 3,62E-05 | NS | 0,016 | 1,95 | up | 1,23 | down |
| 20 | PPP1R3C | 5507_at | 3,62E-05 | NS | 0,017 | 2,21 | down | 1,14 | up |
| 21 | THRSP | 7069_at | 3,65E-05 | NS | NS | 1,64 | down | 1,54 | down |
| 22 | IDI1 | 3422_at | 4,92E-05 | NS | 0,044 | 1,67 | down | 1,13 | up |
| 23 | FGF2 | 2247_at | 5,29E-05 | 0,017 | 2,9E-03 | 2,30 | up | 1,09 | down |
| 24 | LOC55908 | 55908_at | 5,57E-05 | NS | NS | 2,62 | down | 2,03 | down |
| 25 | CDKN1A | 1026_at | 6,39E-05 | 2,2E-03 | 4,8E-03 | 3,49 | down | 1,20 | down |
| 26 | MID1IP1 | 58526_at | 7,40E-05 | 0,029 | 0,020 | 2,23 | down | 1,01 | down |
| 27 | C4orf42 | 92070_at | 7,47E-05 | NS | 0,022 | 2,09 | down | 1,07 | down |
| 28 | ACSS2 | 55902_at | 7,72E-05 | NS | NS | 1,66 | down | 2,16 | down |
| 29 | HLX | 3142_at | 8,23E-05 | NS | NS | 2,30 | down | 1,47 | down |
| 30 | BBS12 | 166379_at | 8,42E-05 | NS | NS | 1,67 | up | 1,10 | down |
| 31 | AACS | 65985_at | 8,60E-05 | NS | NS | 1,84 | down | 2,18 | down |
| 32 | TMEM70 | 54968_at | 8,66E-05 | NS | 1,8E-03 | 2,89 | down | 1,25 | up |
| 33 | NR1D2 | 9975_at | 9,99E-05 | NS | 0,031 | 2,06 | up | 1,00 | - |
| 34 | HEXIM1 | 10614_at | 1,00E-04 | 0,025 | 0,018 | 1,02 | down | 2,43 | up |
| 35 | INSIG1 | 3638_at | 1,11E-04 | NS | NS | 1,46 | down | 1,11 | up |
| 36 | HMGCS1 | 3157_at | 1,18E-04 | NS | NS | 2,02 | down | 1,07 | up |
| 37 | IRF8 | 3394_at | 1,55E-04 | NS | 0,017 | 1,04 | up | 2,31 | up |
| 38 | CTGF | 1490_at | 1,58E-04 | 0,021 | NS | 1,57 | up | 2,90 | up |
| 39 | SLC2A3P1 | 100128062_at | 1,64E-04 | 2,7E-03 | 4,9E-03 | 2,38 | down | 1,18 | up |
| 40 | APOC1 | 341_at | 1,89E-04 | NS | 5,7E-03 | 3,41 | down | 1,04 | up |
| 41 | ACLY | 47_at | 1,97E-04 | NS | NS | 2,99 | down | 2,21 | down |
| 42 | URB2 | 9816_at | 2,01E-04 | NS | 0,012 | 2,66 | down | 1,11 | up |
| 43 | C7orf63 | 79846_at | 2,07E-04 | NS | NS | 1,57 | up | 1,63 | down |
| 44 | SLC31A2 | 1318_at | 2,66E-04 | 4,4E-03 | NS | 2,43 | up | 1,83 | up |
| 45 | GYS2 | 2998_at | 2,80E-04 | NS | NS | 2,81 | down | 4,83 | down |
| 46 | PER1 | 5187_at | 3,07E-04 | NS | NS | 1,20 | up | 1,41 | down |
| 47 | PRUNE2 | 158471_at | 3,07E-04 | 0,017 | 0,044 | 4,40 | down | 1,23 | down |
| 48 | RBM20 | 282996_at | 3,61E-04 | 0,046 | 0,015 | 9,53 | down | 1,78 | down |
| 49 | SPON2 | 10417_at | 3,76E-04 | NS | 6,1E-03 | 1,84 | down | 1,20 | up |
| 50 | KIAA0922 | 23240_at | 4,38E-04 | NS | 0,027 | 2,03 | up | 1,14 | down |
| 51 | IER2 | 9592_at | 5,03E-04 | NS | NS | 1,13 | down | 1,00 | - |
| 52 | LONRF1 | 91694_at | 5,06E-04 | 0,020 | 0,022 | 2,62 | up | 1,00 | - |
| 53 | TLR1 | 7096_at | 5,17E-04 | 0,022 | NS | 3,27 | up | 2,84 | up |
| 54 | PPP1R3B | 79660_at | 5,31E-04 | 2,7E-03 | 0,013 | 4,76 | down | 1,07 | down |
| 55 | RASD1 | 51655_at | 6,05E-04 | NS | NS | 1,36 | up | 1,43 | down |
| 56 | TMEM100 | 55273_at | 6,10E-04 | NS | NS | 1,05 | down | 1,62 | down |
| 57 | ADSSL1 | 122622_at | 6,23E-04 | 0,044 | NS | 2,75 | down | 2,44 | down |
| 58 | LDLR | 3949_at | 6,41E-04 | NS | NS | 1,14 | up | 1,01 | down |
| 59 | OAS1 | 4938_at | 7,46E-04 | NS | NS | 1,42 | down | 1,03 | down |
| 60 | DMRT2 | 10655_at | 7,71E-04 | NS | NS | 1,68 | down | 2,74 | down |
| 61 | ELOVL6 | 79071_at | 8,09E-04 | NS | 0,043 | 6,03 | down | 1,73 | down |
| 62 | LOXL1 | 4016_at | 8,44E-04 | NS | 8,8E-03 | 1,43 | down | 1,83 | up |
| 63 | DUSP4 | 1846_at | 8,51E-04 | 0,010 | NS | 3,68 | down | 2,46 | down |
| 64 | LSS | 4047_at | 8,91E-04 | NS | NS | 1,91 | down | 1,41 | down |
| 65 | SH3RF3 | 344558_at | 9,21E-04 | NS | NS | 1,09 | down | 1,58 | up |
| 66 | ZBTB16 | 7704_at | 1,13E-03 | NS | NS | 2,19 | up | 1,02 | up |
| 67 | FAM49A | 81553_at | 1,13E-03 | 0,016 | NS | 3,28 | down | 1,17 | down |
| 68 | FSTL3 | 10272_at | 1,18E-03 | NS | NS | 1,26 | down | 1,22 | up |
| 69 | SMYD4 | 114826_at | 1,38E-03 | NS | 0,011 | 1,47 | up | 1,14 | down |
| 70 | LRRN4CL | 221091_at | 1,44E-03 | NS | 0,021 | 2,36 | down | 1,06 | down |
| 71 | MAP2K6 | 5608_at | 1,48E-03 | NS | NS | 2,30 | down | 1,09 | down |
| 72 | GADD45A | 1647_at | 1,49E-03 | 0,029 | 0,028 | 2,54 | down | 1,37 | down |
| 73 | PPP1R10 | 5514_at | 1,85E-03 | NS | NS | 1,62 | down | 1,36 | up |
| 74 | HMOX1 | 3162_at | 2,02E-03 | 2,4E-03 | NS | 2,47 | up | 2,56 | up |
| 75 | BMP2 | 650_at | 2,04E-03 | 1,8E-03 | 0,014 | 5,69 | up | 1,97 | up |
| 76 | CTPS | 1503_at | 2,10E-03 | NS | 0,012 | 2,03 | down | 1,93 | up |
| 77 | LPIN1 | 23175_at | 2,39E-03 | NS | NS | 1,41 | down | 1,94 | down |
| 78 | KDM3A | 55818_at | 2,48E-03 | 0,013 | NS | 2,09 | up | 1,00 | - |
| 79 | TPST2 | 8459_at | 2,51E-03 | NS | NS | 1,49 | up | 1,85 | up |
| 80 | SGEF | 26084_at | 2,71E-03 | NS | NS | 1,12 | down | 1,86 | down |
| 81 | MOGAT1 | 116255_at | 2,73E-03 | NS | NS | 2,24 | down | 2,56 | down |
| 82 | CSN1S1 | 1446_at | 2,79E-03 | NS | NS | 2,45 | up | 2,30 | up |
| 83 | FADS1 | 3992_at | 2,83E-03 | NS | NS | 1,78 | down | 2,08 | down |
| 84 | KIF2A | 3796_at | 2,88E-03 | NS | 0,041 | 1,68 | up | 1,18 | down |
| 85 | LIAS | 11019_at | 3,21E-03 | NS | NS | 1,18 | down | 2,24 | down |
| 86 | TSLP | 85480_at | 3,51E-03 | NS | NS | 1,29 | up | 1,48 | down |
| 87 | GFOD1 | 54438_at | 3,66E-03 | 0,017 | 0,013 | 2,73 | down | 1,23 | down |
| 88 | NEU1 | 4758_at | 3,81E-03 | NS | NS | 2,03 | up | 2,24 | up |
| 89 | C10orf10 | 11067_at | 3,89E-03 | NS | NS | 1,40 | up | 1,69 | down |
| 90 | SLA | 6503_at | 3,96E-03 | 5,1E-03 | NS | 2,51 | up | 3,20 | up |
| 91 | MYC | 4609_at | 3,98E-03 | 0,022 | NS | 2,21 | down | 1,03 | down |
| 92 | C11orf48 | 79081_at | 4,07E-03 | NS | NS | 2,71 | down | 1,06 | down |
| 93 | ANGPTL4 | 51129_at | 4,17E-03 | NS | NS | 2,53 | up | 1,60 | down |
| 94 | AVPI1 | 60370_at | 4,22E-03 | NS | NS | 1,32 | down | 1,93 | down |
| 95 | CCL8 | 6355_at | 4,34E-03 | NS | NS | 3,35 | up | 1,31 | up |
| 96 | RAB7L1 | 8934_at | 4,45E-03 | NS | NS | 1,80 | up | 1,87 | up |
| 97 | ELOVL3 | 83401_at | 4,62E-03 | NS | NS | 2,70 | down | 2,71 | down |
| 98 | PTGER2 | 5732_at | 4,68E-03 | NS | NS | 1,32 | up | 1,04 | down |
| 99 | HMGCR | 3156_at | 4,77E-03 | NS | NS | 1,79 | down | 1,26 | up |
| 100 | TTC25 | 83538_at | 4,90E-03 | NS | 0,048 | 4,42 | down | 1,47 | down |
| 101 | PPAPDC2 | 403313_at | 5,09E-03 | 0,041 | 0,024 | 2,40 | up | 1,14 | up |
| 102 | SEL1L2 | 80343_at | 5,17E-03 | NS | 0,011 | 1,54 | down | 2,33 | up |
| 103 | TTC32 | 130502_at | 5,46E-03 | NS | 4,1E-03 | 2,53 | up | 1,21 | up |
| 104 | RWDD2A | 112611_at | 5,52E-03 | NS | NS | 1,81 | up | 1,60 | down |
| 105 | ATF7IP2 | 80063_at | 5,85E-03 | NS | NS | 1,87 | up | 1,22 | up |
| 106 | LGALS12 | 85329_at | 5,92E-03 | NS | NS | 1,42 | down | 1,54 | down |
