# Supplementary material for: Adipose tissue gene expression analysis reveals changes in inflammatory, mitochondrial respiratory and lipid metabolic pathways in obese insulin-resistant subjects
Source: BMC Med Genomics. 2012 Apr 3;5:9. doi: 10.1186/1755-8794-5-9 (PMC3384471; doi:10.1186/1755-8794-5-9)
Supplement: Additional file 5 — Relative mRNA levels of selected genes in mitochondrial respiratory chain pathway. Expression levels were analyzed in insulin-resistant (black bar) and insulin-sensitive subjects (white bar) at 0 h, 3 h, and 6 h during euglycaemic hyperinsulinaemia. All genes are differentially expressed between insulin-resistant and insulin-sensitive groups: p(group) < 0.05, p(group*insulin) = NS, p(insulin) = NS for all genes via 2-way ANOVA. [file 1755-8794-5-9-S5.PPT]

## Slide 1
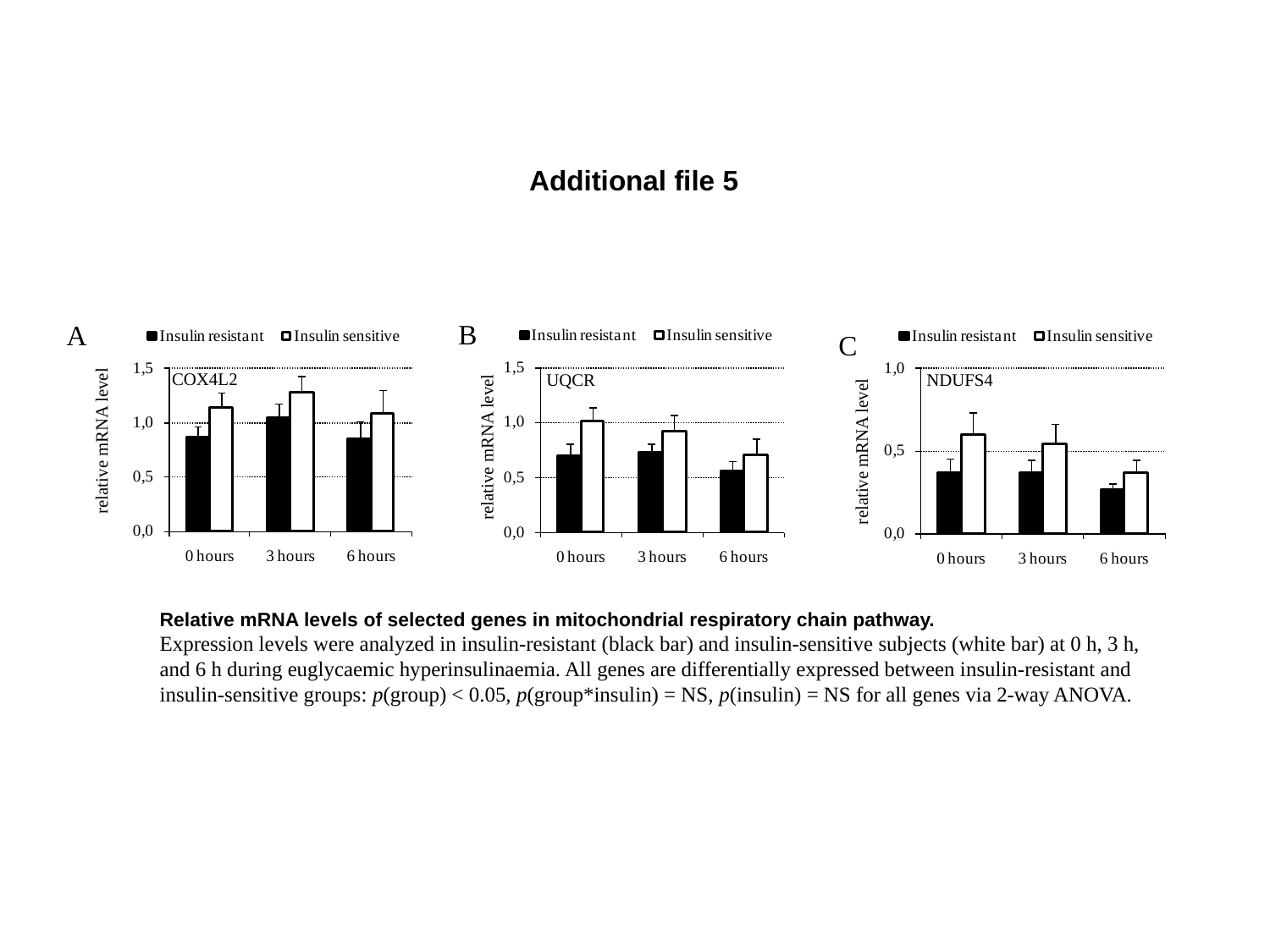

Additional file 5
B
UQCR
A
COX4L2
NDUFS4
C
relative mRNA level
relative mRNA level
relative mRNA level
Relative mRNA levels of selected genes in mitochondrial respiratory chain pathway.
Expression levels were analyzed in insulin-resistant (black bar) and insulin-sensitive subjects (white bar) at 0 h, 3 h, and 6 h during euglycaemic hyperinsulinaemia. All genes are differentially expressed between insulin-resistant and insulin-sensitive groups: p(group) < 0.05, p(group*insulin) = NS, p(insulin) = NS for all genes via 2-way ANOVA.
